# Understanding the Evolutionary Adaptations and the Associated Functional Dynamics of Diatom *Cyclotella Cryptica*: A Chloroplast Genome-wide Comparative Study

**DOI:** 10.2174/0113892029458363260416055201

**Published:** 2026-04-21

**Authors:** Pankaj Kumar Singh, Baskaran Srinivasan, M. Arockia Babu, Nisha Bansal, Thakur Gurjeet Singh, Yogita Tyagi, Roshan Kumar

**Affiliations:** 1 Department of Biotechnology, University Institute of Biotechnology, Chandigarh University, Mohali, 140 413, Punjab, India;; 2 University Centre for Research and Development, Chandigarh University, Mohali, 140 413, Punjab, India;; 3 Department of Microbiology, The Oxford College of Science, Bengaluru, Karnataka 560102, India;; 4 Institute of Pharmaceutical Research GLA University, Mathura 17 Km Stone, National Highway 2, Delhi-Mathura Road, P.O. Chaumuha, Mathura-281406, Uttar Pradesh, India;; 5 Department of Biotechnology, Graphic Era (Deemed to be University) Clement Town Dehradun- 248002, India;; 6 Centre for Research Impact & Outcome, Chitkara College of Pharmacy, Chitkara University, Rajpura, 140401, Punjab, India;; 7 Uttaranchal Institute of Pharmaceutical Sciences, Uttaranchal University, Premanagar, Dehradun 248007, Uttarakhand, India;; 8 Department of Microbiology, Central University of Punjab, Ghudda, Bathinda, Punjab, India

**Keywords:** cpDNA, cyclotella, chloroplast, biofuel, diatoms, cyclotella cryptica, comparative analysis

## Abstract

**Introduction:**

*Cyclotella cryptica* is a marine diatom recognized as a promising biofuel resource and a model organism for studying diatom physiology and biotechnology. Since lipid biosynthesis in microalgae initiates in plastids, understanding chloroplast genome organization is crucial for advancing biofuel-related research.

**Objective:**

The work is focused on sequencing and analyzing the chloroplast genome of C. cryptica and performing a phylogenomic comparative analysis.

**Methods:**

The sequencing and annotation of the chloroplast genome of *C. cryptica* were performed. Comparative phylogenomic analysis was conducted using 27 diatom chloroplast genomes to investigate evolutionary relationships.

**Results:**

The chloroplast genome was 129,273 bp in length and contained 132 predicted gene biotypes, including 57 protein-coding genes, 67 tRNA genes, and 8 rRNA genes. Phylogenomic analysis revealed that centric diatoms clustered into a single clade, whereas pennate diatoms formed two distinct clades.

**Discussion:**

The conserved clustering of centric diatoms and the clear separation from pennate diatoms reflect established evolutionary patterns within diatoms and highlight the conserved nature of chloroplast genomes in Cyclotella species.

**Conclusion:**

These findings enhance understanding of chloroplast genome organization and evolution in Cyclotella and other diatoms and provide valuable genomic insights that may support future research aimed at improving biofuel production.

## 1 INTRODUCTION

In the primary endocytobiosis process, a unicellular heterotrophic eukaryote ingested a photosynthetic cyanobacterium, transforming it into a chloroplast [1, 2]. All terrestrial plants are descended from the three fundamental ancestries of eukaryotic algae known as Rhodophyta (red algae phylum), glaucophytes (freshwater algae phylum), and Chlorophyta (green algae phylum), which were the result of primary endocytosis [3]. As much as 1.6 billion years ago, this first endocytobiosis event may have occurred [4], and was a critical phase in the evolution of eukaryotic cells. At least two instances of secondary endocytobiosis led to a significant increase in algal diversity. When a rhodophyte was incorporated by a heterotrophic flagellate (Oomycetes), all Chromists, including Cryptophyta, Dinophytes, Haptophytes, and Stramenopiles, evolved. On the other hand, integrating a green alga within a eukaryotic host cell resulted in the emergence of dinophytes, euglenoids, and chlorarachniophytes (Heterokontophyta) [5-8]. Secondary endocytosis might have occurred 1.3 billion years ago [4]. The outermost membrane of the four-membrane plastids is present in all Cryptophyta and heterokontophyte algae (diatoms, brown algae, and similar algae), and includes endoplasmic reticulum (ER) membrane systems that may be continuous [9, 10]; therefore, it is known as CER (chloroplast endoplasmic reticulum) [11].

The two main plastid lineages produced by the primary endosymbiosis, the rhodoplasts and chloroplasts, fundamentally differ from the complex or secondary plastids of diatoms. Higher plants and green algae have chloroplasts with stacked thylakoid membranes and auxiliary pigments, such as chlorophyll a and b. Rhodoplasts, on the other hand, are found in red algae and capture light energy through phycobilisomes and chlorophyll a. The photosynthetic thylakoid membrane structure in diatom plastids is reminiscent of that in heterokonts. The thylakoids are arranged in sets of three lamellae, each surrounded by a girdle lamella [1]. Diatoms' photosynthetic system also includes a standard set of pigments. Chlorophylls a and c and the carotenoid fucoxanthin, which gives plants their brown hue, are all found in the plastids [12]. There are hints that the diatoms might be able to perform C4 photosynthesis, which would add another justification for their biological success in the planet's oceans [13].

A plastid is an organelle found in algae and plants that contains a small genome (ptDNA or plastome). Along with mitochondria, both possess similar eubacterial features, such as operon gene structure, the prokaryotic mode of gene transcription, and the translation process. The plastome is a circular, double-stranded DNA molecule that is highly polyploid, ranging in size from 120 to 180 kb and containing approximately 120 genes. A duplication event involving roughly 25 kb in inverted orientation, known as “inverted repeats,” is a notable feature of the plastid genome [14-17].

Diatoms, which originated in the Stramenopiles, trace their roots to the Cretaceous period, which spanned 144 to 165 million years ago (Mya). [18]. They might even have a 550-million-year evolutionary history ^3^ with over 250 genera and 200,000 species [19-21]. As in other findings, it has been determined that diatoms appeared 188 Mya, based on divergence-time estimates [22]. The Horizontal Gene Transfer (HGT) technique is employed for the incorporation of several genes into the organelle and nuclear genomes. Moreover, the endosymbiosis technique has transferred substantial gene-related machinery into the genomes of algae *via* endosymbiotic gene transfer (EGT). In the nuclear genomes of diatoms such as Phaeodactylum tricornutum Bohlin, 1897 (a model diatom) and Thalassiosira pseudonana (Hustedt) Hasle & Heimdal, 1970, many hundreds of genes are reported to have been acquired from diverse bacterial lineages through HGT [23, 24]. The sequenced diatom chloroplast genomes exhibit high levels of similarity. However, there are known instances of the replacement or introduction of a gene *via* HGT [25]. Consequently, greater chloroplast genome sequence similarity between the diatom Nitzschia palea (Kützing) W. Smith, 1856 and the dinoflagellate Durinskia baltica (Levander) Carty & E.R.Cox, 1986 indicates that the former was an endosymbiont [26].

Plasmid genomes have been widely employed in functional genomics, facilitating activities such as gene knockouts, overexpression experiments, and specific site-directed mutagenesis of plastid genes. Due to advances in next-generation DNA sequencing technologies, numerous chloroplast genomes (cpDNAs) of algae have been sequenced and are now being used as a next frontier of molecular markers. These genomes harbor ample variations that enable the differentiation of closely related species and strains [27, 28]. The cpDNAs are commonly referred to as “super-barcodes” due to their significantly longer sequences than typical molecular markers, their extensive genetic information, and the presence of numerous PCGs (protein-coding genes) [29-32]. *Thalassionema* cpDNAs have conserved gene content both within and between species; a comparative study reported multiple gene loss and transfer events. In addition, unlike other diatom cpDNAs, it includes extended Inverted Repeat (IR) sequence regions and huge intergenic spacer sequences. Approximately 69 million years ago, *Thalassionema species* separated from *Synedra acus* Kützing, 1844, and roughly 38 million years ago, the *Thalassionema species* diverged, forming a monophyletic clade [33].

Moreover, genes encoding plastid proteins align easily across various diatoms [34]. The comparison of chloroplast genomes of Halamphora and the land plants reveals that this genus's diatoms may be progressing much faster, 4–7 times faster than the land plants, in terms of the chloroplast genome [35]. When it comes to classifying and identifying species and researching the intricate evolutionary relationships among algal species, cpDNA has been extensively employed as a source of vital information for the in-depth understanding of plant evolutionary biology [36, 37]. Using cpDNAs as superbarcodes to differentiate among various plant species has been proposed, and the chloroplast genome sequences of the Bacillariaceae family exhibit substantial variation, supporting this hypothesis [38].

## 2 MATERIALS AND METHODS

The DNA extraction and purification from the Chlorophyta Cyclotella cryptica Reimann, Lewin & Guillard, 1963 chloroplasts were performed with the help of Plasmid Mini Kit (Qiagen India Pvt. Ltd.) following the user manual from the manufacturer. Additionally, the time period of cell lysis was adjusted to 1 h of incubation at 37 °C with continuous shaking (gentle) throughout the lysis process. The isolated DNA was resuspended in 20 μL of 1 mM Tris–HCl buffer (pH 8.0) and transferred to storage conditions set at 4 °C for future experiments. Quantification and purity of isolated DNA were determined *via* recording absorbance at 260 nm with a NanoDrop instrument (Thermo Fisher Scientific India Pvt. Ltd.). The 260/280 ratio was calculated based on the absorbance readings.

### 2.1 Chloroplast Genome Sequencing and Assembly

The sequencing of purified chloroplast DNA (cpDNA) from Cyclotella cryptica was achieved with the help of the 454/Roche FLX high-throughput sequencing platform (Roche, Basel, Switzerland). The acquired Sff-file underwent preprocessing, which involved trimming of poor-quality sequences. In preprocessing analysis, SFF raw sequencing data were converted into fastq format using a Python script, sff2fastq.py. The data quality check and trimming analyses were performed by FastQC tool version 0.11.8 and BBMap version 38.87, respectively. The reference-based assembly was performed on the preprocessed clean data using the BWA sequence aligner version 0.7.17-r1188 [39]. In this assembly analysis, a chloroplast genome (cp genome) sequence of *Cyclotella cryptica* was used as the reference genome [40]. The positions and directions of the aligned sequences were predicted from the reference sequence. The reference chloroplast genome sequence was retrieved from the website http://genomes.mcdb.ucla.edu/Cyclotella/CcrChrChd03da03.fasta. The alignment Sam file was further converted to a Bam file using Samtools version 1.13 [41], and the Bam file was then sorted by position using Samtools. The consensus sequences were retrieved from the sorted BAM alignment file using a Perl script, vcfutils.pl bundled with Bcftools along with seqtk version 1.3-r117-dirty. The consensus sequences were used in all downstream analyses.

### 2.2 Gene Prediction and Annotation Analysis

The newly acquired complete chloroplast genome sequence was used for annotation analysis using GeSeq, version 2.03 [42]. The following parameters were chosen for the annotation analysis: plastid (algae) as the sequence source; support annotation by Chloe version 0.1.0; keep only the best annotation; ARAGORN version 1.2.38 [43] with bacterial/plant chloroplast; and tRNAscan-SE version 2.0.7 [44] with organellar tRNAs. Further, slight gene coordinate adjustments were made based on the start and stop codons using Sequin version 10.2 The revised cp genome sequence was finally submitted to the GenBank repository of the National Center for Biotechnology Information. For this submission, MT385839 was the accession number obtained by GenBank. Another web tool, CPGAVAS2, was also utilized for the cp genome annotation [45].

### 2.3 Circular Physical Map Construction

Converting the sequence into GenBank format facilitated the creation of a physical map of the chloroplast genome and the subsequent submission of the resulting information to GeSeq [42], CPGAVAS2 [45], and GenomeVx (http://wolfe.ucd.ie/GenomeVx/) for online analyses [46].

### 2.4 Repeats Detection

Simple Sequence Repeat (SSR) loci were detected using the MISA tool within the CPGAVAS2 package [45]. In this SSR analysis, the following parameter was considered: Mononucleotides ≥8 repeat units; di- and tri-nucleotides ≥4 repeat units, and ≥3 repeat units for tetra, penta-, and hexa-nucleotides. An independent SRR prediction analysis was also carried out to verify the MISA results using Krait v.1.3.2, with the same parameters as in the MISA analysis [47]. For the identification of long, complex repeats and long tandem repeats, the REPuter module of Vmatch [48] and the Tandem Repeats Finder (TRF) tool version 4.09 [49] were used, which is available within the CPGAVAS2 annotation analysis [45]. The minimum repeat length was set to 30 bp, along with a Hamming distance of 3 for the long, complex repeat analysis, reverse, forward, palindromic, and complement analyses. The analysis was set to report the best matches with the smallest E-values, with a default limit of the top 50 results. Moreover, the repeat unit size was fixed at ≥7 bp in this repeat analysis.

### 2.5 Phylogenomic Comparative Analysis

The phylogenetic analysis was performed using 27 cp genomes, including the cp genome currently under study (Table **S1**). A multiple sequence alignment (MSA) was performed with MAFFT version 7.453 [50]. The MSA analysis was conducted with auto mode. Further, the MSA file was analyzed with IQ-TREE version 1.6.12 to predict the phylogenetic model and generate a tree [51]. The IQ-TREE tool uses a rapid, efficient stochastic algorithm to infer phylogenetic trees *via* maximum likelihood. The best model prediction and model selection during tree generation were automatically carried out by IQ-TREE. In this phylogenetic tree construction analysis, standard model selection followed by tree inference, ultrafast bootstrap values (1000), and SH-like aLRT (approximate likelihood ratio test) based on 1000 replicates were used as parameters.

## 3 RESULTS AND DISCUSSION

### 3.1 Chloroplast Genome Sequencing, Assembly, and Annotation

The chloroplast (cp) genome of a strain of *Cyclotella cryptica* was sequenced and annotated. The chloroplast genome comprises four discrete sections: two inverted repeat regions, IRA and IRB, a large single copy region (LSC), and a small single copy region (SSC) (Fig. **1**). The complete chloroplast genome size was determined to be 129,273 bp (Fig. **1**). The length of SSC, LSC, IRA, and IRB was found to be 3,0884 bp (81,814-112,697), 61,052 bp (66,730-127,781), 15,083 bp (112,698-127,780), and 15,083 bp (66,731-81,813), respectively. Annotation analysis of the cp genome resulted in 132 gene biotypes, including 57 protein-coding genes, 67 tRNAs, and 8 rRNAs (Table **1**). A total of 132 genes identified in the gene annotation analysis were grouped into three biological process groups: the protein synthesis and DNA-replication group, the photosynthesis group, and the miscellaneous group. Gene density was estimated at 1021.09 genes per megabase (Mb) using the formula: total gene count divided by the sequence length in megabases. The rRNA and tRNA genes were further categorized into ribosomal and transfer gene types within the protein synthesis and DNA replication biological processes. This biological process also included ribosomal protein genes (large and small subunit) and RNA polymerase genes. The miscellaneous group had a single gene related to Cytochrome c biogenesis. The photosynthesis biological process is categorized into seven gene types: Photosystem I, Cytochrome b/f complex, Photosystem II, Rubisco large subunit, ATP synthase, Photosystem biogenesis factor, and Photosystem assembly protein ycf (Table **1**). The highest percentage of genes was found for the transfer RNA gene type (50.76%), followed by the photosystem II (9.85%) and the ribosomal protein genes, both the large and small subunits (7.58%). Genes involved in photosynthesis may be directly associated with high photosynthetic efficiency, leading to increased lipid accumulation and, consequently, enhanced biofuel and omega-3 fatty acid production. Therefore, decoding additional *C. cryptica* genomes—whether chloroplast, mitochondrial, or nuclear —would aid more effective metabolic engineering of these processes and further support its development as a genetically tractable organism [40].

### 3.2 Identification of Repeat Sequences

A total of 129,273 nucleotide bases in a single stretch sequence were analyzed to identify repeats. A total of 48 long repeats were determined in this cp genome sequence (Supplementary Table **1**). However, the cp genome contained only three long tandem repeats, each with a repeat unit size of 7 or greater. The cp genome sequences 22,478-22,520, 26,902-26,956, and 65,139-65,304 were detected as long tandem repeats. Approximately 4.47% of nucleotides were masked and identified as different types of repeats. It consisted of 0.15% interspersed repeats, 2.92% small RNA, 1.13% simple repeats, and 0.42% low complexity regions. However, 59.3% (101.5 Mb) of genomic regions were characterized as repeats in *C. cryptica* [40].

SSRs have been used as DNA molecular markers to estimate variability within related species and are useful for investigating population genetics [52]. 118 perfect SSRs were detected in the cp genome currently studied, accounting for 0.86% of the cp genome (Table **2**).

A similar SSR prediction study in 11 chloroplast genomes of different algae was done by Cai *et al.* [53] and reported that a minimum of 92 and a maximum of 869 SSRs were detected from *Ostreobium sp.* (KU979013) and *Gloeotilopsis sp.* (KX306821), respectively. Thus, the 118 total SSRs predicted in the current study fall within the range reported by Cai *et al.* [53]. As expected, 81 counts were obtained for the mono-nucleotide SSR motif, followed by Di-, Tetra-, Tri-, Penta-, and Hexa-nucleotide motifs (Supplementary Fig. **S1**). A similar trend of SSR repeat counts was also observed by Cai *et al.* in *Ulva flexuosa* Wulfen, 1803 cp genomes [53]. However, maximum relative density (bp/Mb) was obtained for motif “A” followed by “AT,” “AATT,” “And AC,” while minimum relative density was found for “AAG,” “AAT,” and “ACG” (Supplementary Table **2**; Supplementary Fig. **S2**). It has been reported that the mononucleotide type repeats contain “A/T” motif sequences in chloroplast genomes [53]. Mononucleotide SSRs had the highest distribution (62%) among SSR motif types based on sequence length across the current Cyclotella cp genome. In contrast, hexanucleotide SSR showed the lowest length distribution in this cp genome (Supplementary Fig. **3**). Our results align with earlier reports, which found that SSRs in cp genomes are mainly short polyp or poly (A) repeats and rarely comprise C or G repeat types [53, 54].

### 3.3 Phylogenetic Relationship of Chloroplast Genomes

A total of 27 complete chloroplast (cp) genome sequences, including the currently studied chloroplast genome, were used to infer the genetic relationships among the algae. The best-suited model, GTR+F+I+G4, was identified based on Akaike Information Criterion (AIC) scores and Bayesian Information Criterion (BIC) score rankings. AIC is a mathematical approach for estimating how fine a model fits the subject data. It generally compares different likely models and determines which is the top-fitting data model. BIC also does the same thing to find the best model fit to the subject data, but uses a different mathematical method: Bayesian probability.

Based on the phylogenetic inferred tree derived by the phylogenetic analysis using the chloroplast genome of diatoms, the centric diatoms were grouped in a single clade, whereas the pennate diatoms were grouped in two separate clades (Fig. **2**). *Asterionella formosa* Hassall, 1850 (NC_024079.1), *Asterionellopsis glacialis* (Castracane) Round, 1990 (NC_024080.1), and *Cylindrotheca closterium* (Ehrenberg) Reimann & J.C.Lewin, 1839 (NC_024082.1) [32] were the three exceptional pennate diatoms included in the clade of centric diatoms. Simultaneously, *Odontella sinensis* (Greville) Grunow, 1884 (NC_001713.1) [55] was the only centric diatom placed away from the centric diatom clade. *Cyclotella cryptica* (MT385839) was grouped with other *Cyclotella species* (KJ958480.1 and KJ958481.1) in a separate clade [56], which was found closely associated with *Thalassiosira pseudonana* (EF067921.1 and NC_008589.1) [57], *Thalassiosira weissflogii* (Grunow) G. Fryxell & Hasle, 1977 (NC_025314.1) [56], *Thalassiosira oceanica* Hasle, 1983 (GU323224.1 and NC_014808.1) [58] and *Roundia sp.* (KJ958483.1 and NC_025312.1) [56] forming an individual clade. Simultaneously, Sabir *et al.* [56] reported close collinearity between the chloroplast genomes of Cyclotella, Skeletonema, and Thalassiosira and those of *Roundia*.

The chloroplast genome of a pennate diatom, Seminavis robusta D.B.Danielidis & D.G.Mann, 2002 (MH356727.1), was confirmed to have been recently transferred from the Volvox kind of green microalgae in the course of evolution, and it is the largest chloroplast genome with 159,905 bp length [25]. However, another chloroplast genome of a pennate diatom, *Nanofrustulum shiloi (J.J.Lee, Reimer & McEnery) Round, Hallsteinsen & Paasche, 1999 (MN276191.1), was found with 160,994 bp length,* and it forms a separate araphid clade with Synedra, Asterionella, and Licmophora [59]. *Thalassiosira* differed in morphology from Skeletonema and Cyclotella, as reflected in its phylogeny as well [60].

The phylogenomic analysis clearly differentiated centric and pennate diatoms into distinct clades, consistent with their well-established morphological and evolutionary divergence (Fig. **2**). Within the centric diatoms, members of the genus Cyclotella clustered together, indicating a close evolutionary relationship and a high level of chloroplast genome conservation within the genus. This conserved genomic architecture suggests shared evolutionary origins and functional constraints among Cyclotella species. However, the present study is limited by its reliance on chloroplast genome data alone and a restricted number of available Cyclotella genomes. Future studies integrating nuclear and mitochondrial genomes, along with broader taxon sampling, will provide a more comprehensive understanding of evolutionary relationships and functional diversification within diatoms [40].

In an interesting study, the diatom Nitzschia palea and the dinoflagellate *Durinskia baltica* chloroplast genomes showed pairwise sequence similarity exceeding 90%. Hence, chloroplast phylogenies revealed that Durinskia baltica was *Durinskia baltica*’s endocytobiont [26]. Contrastingly, the chloroplast DNA of the genus *Nitzschia* exhibits highly consistent and diverse genome arrangements compared with other genera such as *Chaetoceros, Skeletonema*, and *Thalassiosira* [60-62]. Comparatively, the chloroplast genome of *Thalassionema* shows highly conserved genes at the inter- and intra-species levels, with more gene transfers, losses, and stretched inverted repeat regions (IRs), and thus conserves large intergenic spacers [33]. Gene duplication by inversions, deletions, and insertions in IR and single-copy (SR) regions led to several gene-order changes in diatoms, resulting in a positive correlation between plastome rearrangement and nucleotide substitution rates, similar to that observed in legume plants [63].

## STUDY LIMITATIONS

The comparative analysis solely depends on chloroplast genome (cpDNA) data, *i.e*., only a part of the organism’s genetic system, which limits the applicability of the study. Furthermore, the study needs to be validated with a large dataset (27 chloroplast genomes in this study) with broad taxonomic sampling for robustness. Further studies are also required for experimental functional validation of gene functions, particularly those associated with photosynthesis, lipid metabolism, or biofuel production.

## CONCLUSION

*Cyclotella cryptica* is considered a potential biofuel resource. Sequencing of the chloroplast genome provides molecular insights into chloroplast-encoded genes and pathways that may be indirectly associated with photosynthesis-related processes relevant to biofuel research. However, functional roles in carbon-concentration mechanisms, CO_2_-fixation efficiency, or biofuel production efficiency cannot be inferred solely from chloroplast genome data. In the present study, the complete chloroplast genome of *C. cryptica* was decoded and annotated, yielding a circular genome of 129,273 bp. Phylogenomic analysis revealed the formation of three distinct clades, one comprising centric diatoms and two comprising pennate diatoms. Notably, all four Cyclotella chloroplast genomes clustered into a single subclade, including the genome reported in this study, indicating a high level of chloroplast genome conservation within the genus. While such conservation suggests potential similarities in chloroplast-associated metabolic features, the involvement of specific lipid biosynthesis pathways requires further investigation at the gene and functional levels.

## Figures and Tables

**Fig. (1):**
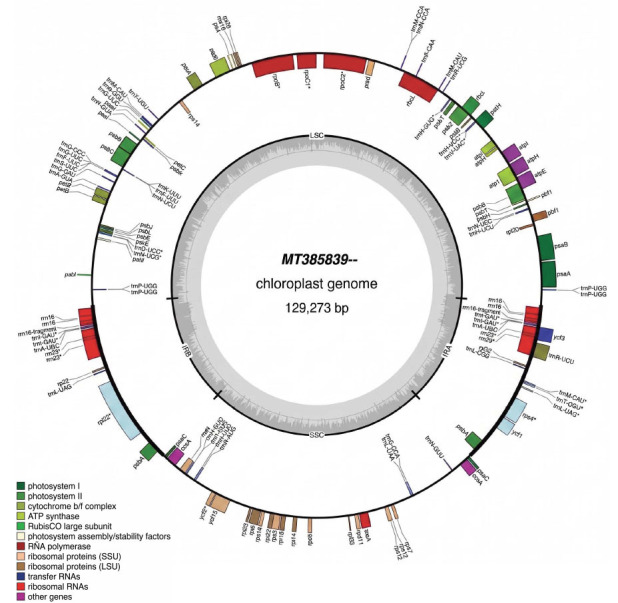
Circular physical map of the Cyclotella chloroplast (cp) genome (Accession no. MT385839). The outer-circle genes undergo clockwise transcription, while the inner-circle genes are transcribed in the opposite direction. Genes categorized into diverse functional groups are represented with distinct color codes. The innermost circle shows the orientation of IRA (Inverted repeat unit), IRB (Inverted repeat unit), small single-copy region (SSC), and a large single-copy region (LSC), in the cp genome.

**Fig. (2):**
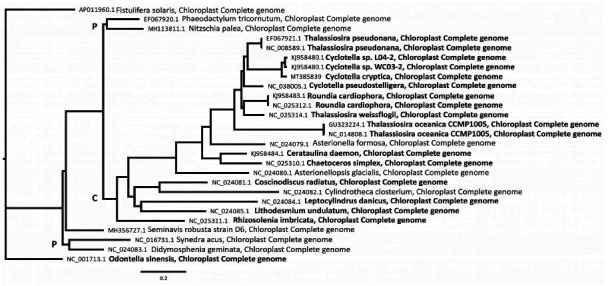
Phylogenetic tree showing the relationship of Cyclotella and other diatoms. This tree was generated from the complete chloroplast genome sequences of 27 marine algae. *Cyclotella* sp. are clustered together along with *Thalassiosira* sp. Two distinct clades of diatoms, pennate and centric, represent “P” and “C,” respectively, at a branch of the tree.

**Table 1:** List of genes annotated in the Cyclotella chloroplast genome.

**Biological Process**	**Gene Type**	**Gene Name**
Protein synthesis and DNA replication	Ribosomal RNA genes	*rrn16*, *rrn23* (8)*
-	Transfer RNA genes	*trnR-UCU*, *trnV-UAC*, *trnR-CCG*, *trnM-CAU*, *trnF-GAA*, *trnW-CCA*, *trnT-UGU*, *trnS-GCU*, *trnD-GUC*, *trnY-GUA*, *trnK-UUU*, *trnG-GCC*, *trnE-UUC*, *trnS-UGA*, *trnG-UCC*, *trnP-UGG*, *trnI-GAU*, *trnA-UGC*, *trnL-UAG*, *trnH-GUG*, *trnQ-UUG*, *trnR-ACG*, *trnN-GUU*, *trnC-GCA*, *trnL-UAA* (67)
-	Ribosomal protein genes (larger subunit)	*rpl2*, *rpl14*, *rpl16*, *rpl20*, *rpl22*, *rpl23*, *rpl32*, *rpl33*, *rpl36* (10)
-	Ribosomal protein genes (smaller subunit)	*rps2*, *rps3*, *rps4*, *rps7*, *rps8*, *rps11*, *rps12*, *rps14*, *rps18*, *rps19* (10)
-	RNA polymerase	*rpoA*, *rpoB*, *rpoC1*, *rpoC2* (4)
Photosynthesis	Photosystem I	*psaA*, *psaB*, *psaC*, *psaI* (5)
-	Photosystem II	*psbA*, *psbB*, *psbC*, *psbD*, *psbE*, *psbF*, *psbH*, *psbI*, *psbJ*, *psbK*, *psbL*, *psbT* (13)
-	Cytochrome b/f complex	*petA*, *petB*, *petD*, *petG*, *petN* (5)
-	ATP synthase	*atpA*, *atpB*, *atpH*, *atpI* (4)
-	Rubisco large subunit	*rbcL* (1)
-	Photosystem biogenesis factor	*pbf1* (1)
-	Photosystem assembly protein ycf	*pafI* (*ycf3*), *pafII* (*ycf4*) (2)
Miscellaneous group	Cytochrome c biogenesis	*ccsA* (2)

**Note:** ()*Total number.

**Table 2:** Information on predicted microsatellites from the Cyclotella chloroplast genome.

**Item**	**Description**	**Number**
Total length of sequences	A+T+C+G (bp)	129,273
GC content	(G+C)/(A+T+C+G) (%)	30.08
Total number of perfect SSRs	Counts	118
Total length of perfect SSRs	Bp	1,103
The average length of SSRs	Total SSR length/total SSR counts (bp)	9.35
SSRs per sequence	Total SSR counts/sequence counts	118
The percentage of the sequence covered by SSRs	Total SSR length/total sequence length (%)	0.86
Relative abundance	Total SSRs/total valid length (loci/Mb)	912.8
Relative density	Total SSR length/total valid length (bp/Mb)	8,532.33

## Data Availability

The authors confirm that the data supporting the findings of this research are available within the article.

## LIST OF ABBREVIATIONS

AIC: Akaike Information Criterion
BIC: Bayesian Information Criterion
CER: Chloroplast Endoplasmic Reticulum
EGT: Endosymbiotic Gene Transfer
ER: Endoplasmic Reticulum
HGT: Horizontal Gene Transfer
MSA: Multiple Sequence Alignment
SSR: Simple Sequence Repeat
TRF: Tandem Repeats Finder
