# Supplementary material for: Understanding the Evolutionary Adaptations and the Associated Functional Dynamics of Diatom Cyclotella Cryptica: A Chloroplast Genome-wide Comparative Study
Source: Curr Genomics. 2026 Apr 21;26(7):833–42. doi: 10.2174/0113892029458363260416055201 (PMC13154245; doi:10.2174/0113892029458363260416055201)
Supplement: Supplementary file 1 [file CG-26-7-833_SD1.pdf]

Supplementary Material

Understanding the Evolutionary Adaptations and the Associated Functional Dynamics of Diatom *Cyclotella Cryptica*: A Chloroplast Genome-Wide Comparative Study

Pankaj Kumar Singh[1,2], Baskaran Srinivasan[3], M. Arockia Babu[4], Nisha Bansal[5,*], Thakur Gurjeet Singh[6], Yogita Tyagi[7] and Roshan Kumar[8,*]

[1]Department of Biotechnology, University Institute of Biotechnology, Chandigarh University, Mohali, 140 413, Punjab, India; [2]University Centre for Research and Development, Chandigarh University, Mohali, 140 413, Punjab, India; [3]Department of Microbiology, The Oxford College of Science, Bengaluru, Karnataka 560102, India; [4]Institute of Pharmaceutical Research GLA University, Mathura 17 Km Stone, National Highway 2, Delhi-Mathura Road, P.O. Chaumuha, Mathura-281406, Uttar Pradesh, India; [5]Department of Biotechnology, Graphic Era (Deemed to be University) Clement Town Dehradun- 248002, India; [6]Centre for Research Impact & Outcome, Chitkara College of Pharmacy, Chitkara University, Rajpura, 140401, Punjab, India; [7]Uttaranchal Institute of Pharmaceutical Sciences, Uttaranchal University, Premanagar, Dehradun 248007, Uttarakhand, India; [8]Department of Microbiology, Central University of Punjab, Ghudda, Bathinda, Punjab, India

Supplementary Table 1. Identification of long repeats in the Cyclotella chloroplast genome.

Repeat Length (part 1)	Start Position (part 1)	Match Direction	Repeat Length (part 2)	Start Position (part 2)	Distance	E-value
6860	66730	P	6860	120920	0	0.00E+00
3670	73591	P	3670	117249	0	0.00E+00
3044	78769	P	3044	112697	0	0.00E+00
1506	77262	P	1506	115742	0	0.00E+00
1491	65238	P	1491	127781	0	0.00E+00
1044	82207	P	1044	111259	-3	0.00E+00
1020	82231	P	1020	111259	-1	0.00E+00
996	82255	P	996	111259	0	0.00E+00
414	81815	P	414	112281	-2	2.02E-234
218	83252	P	218	111040	0	2.65E-122
92	66765	P	92	66765	-2	7.22E-42
92	66765	D	92	127653	-2	7.22E-42
92	127653	P	92	127653	-2	7.22E-42
83	65138	D	83	65221	0	5.02E-41
80	23127	P	80	23127	0	3.22E-39
66	65155	P	66	129206	0	8.63E-31
65	86746	D	65	88750	0	3.45E-30
66	46224	P	66	46224	-2	1.67E-26
59	4677	P	59	4677	-1	2.50E-24
45	66812	D	45	127700	0	3.80E-18
42	85222	P	42	85270	0	2.43E-16

51	6736	P	51	81921	-3	5.21E-16
39	7986	P	39	6736	-1	1.82E-12
35	966	D	35	3275	0	3.98E-12
33	81881	P	33	7986	0	6.37E-11
33	81881	D	33	112556	0	6.37E-11
33	81921	D	33	112596	0	6.37E-11
33	112556	P	33	112596	0	6.37E-11
41	2172	D	41	4475	-3	2.80E-10
38	71581	P	38	71581	-2	3.94E-10
38	71581	D	38	122891	-2	3.94E-10
38	122891	P	38	122891	-2	3.94E-10
30	109457	P	30	109511	0	4.08E-09
36	110641	P	36	110641	-2	5.64E-09
36	1012	D	36	3321	-3	1.92E-07
35	39015	P	35	39015	-3	7.04E-07
34	51196	P	34	51201	-3	2.57E-06
31	64092	P	31	64130	-2	4.27E-06
30	103887	P	30	103888	-2	1.60E-05
32	1818	D	32	4142	-3	3.41E-05
30	26920	P	30	26955	-3	4.47E-04
30	27149	P	30	27153	-3	4.47E-04
30	62513	P	30	62515	-3	4.47E-04
30	72405	P	30	72429	-3	4.47E-04
30	72405	D	30	122051	-3	4.47E-04
30	72429	D	30	122075	-3	4.47E-04
30	106071	P	30	106099	-3	4.47E-04
30	122051	P	30	122075	-3	4.47E-04

P- Palindromic match; D- Direct match

Supplementary Table 2. Abundance of SSR motif category identified from the Cyclotella chloroplast genome.

Motif	Type	Counts	Length (bp)	Percent (%)	Average Length (bp)	Relative abundance (loci/Mb)	Relative density (bp/Mb)
A	1	79	668	66.96	8.46	611.11	5167.36
C	1	2	16	1.69	8.0	15.47	123.77
AG	2	2	16	1.69	8.0	15.47	123.77
AT	2	24	248	20.34	10.33	185.65	1918.42
AAG	3	1	12	0.85	12.0	7.74	92.83
AAT	3	1	12	0.85	12.0	7.74	92.83
ACG	3	1	12	0.85	12.0	7.74	92.83

AAAT	4	1	16	0.85	16.0	7.74	123.77
AATT	4	3	40	2.54	13.33	23.21	309.42
AAAAC	5	1	15	0.85	15.0	7.74	116.03

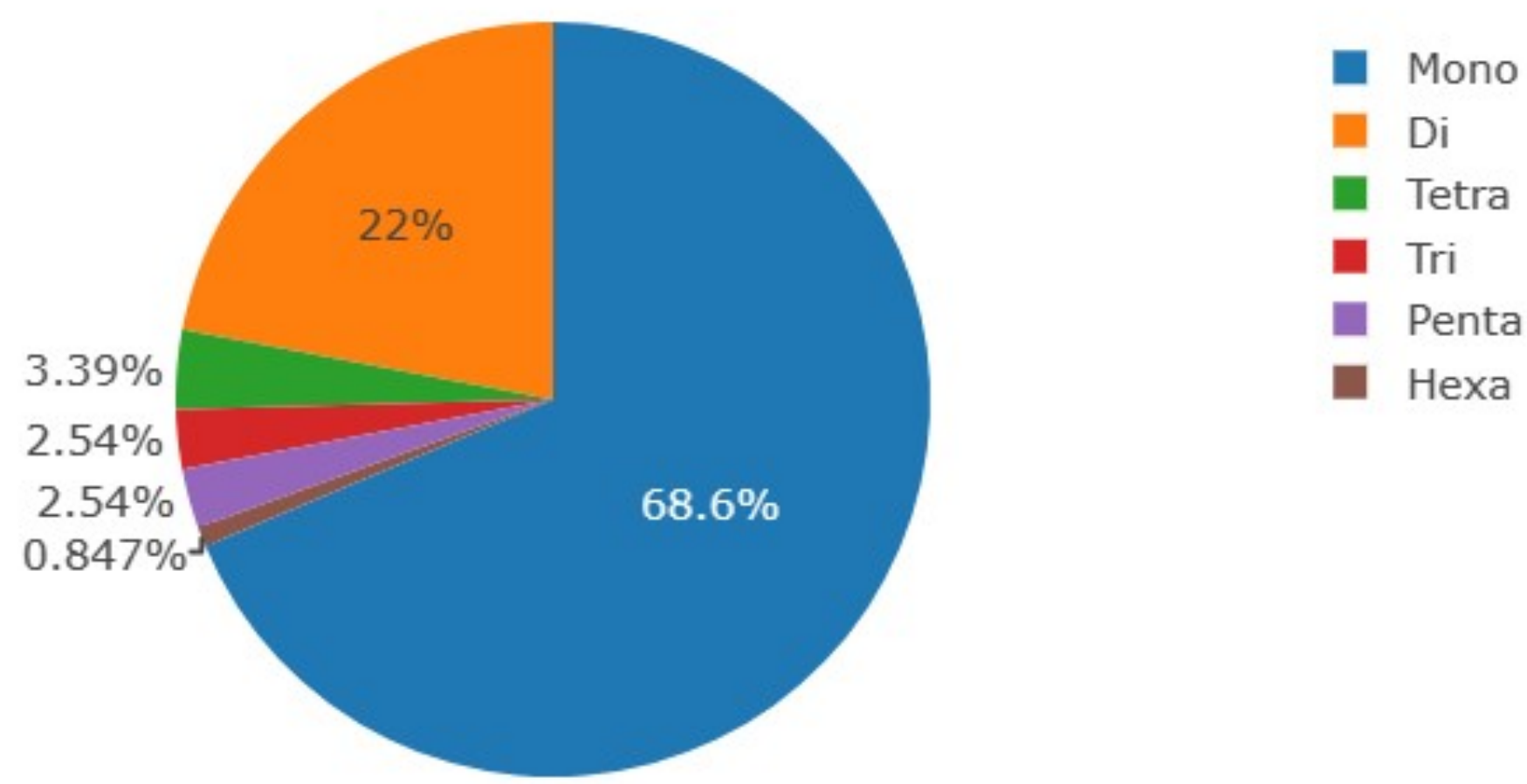

Supplementary Fig. (1). Type of SSR distribution in the Cyclotella chloroplast genome. Different types of SSR based on the type of motif repeats are shown with distinct colours.

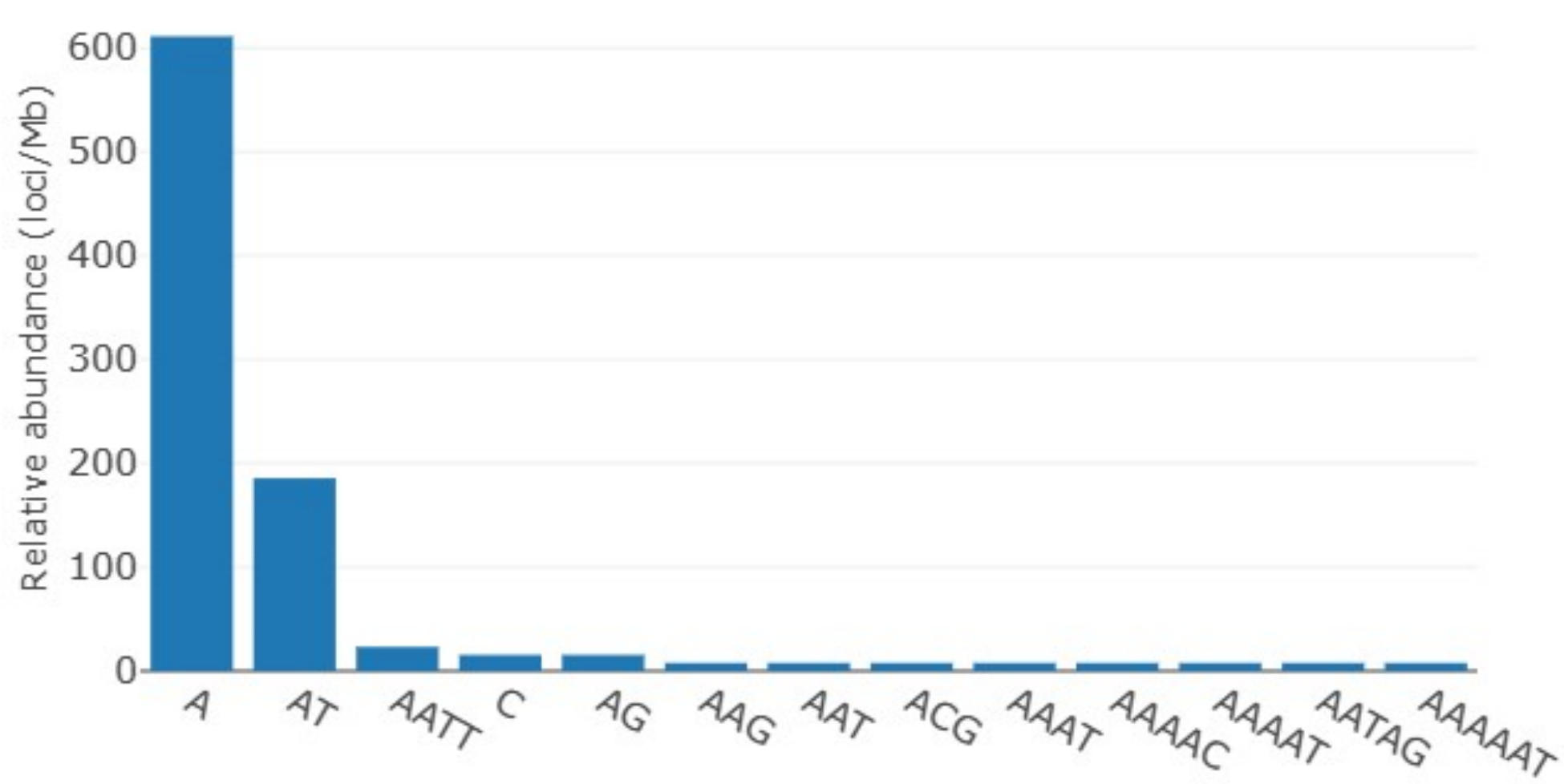

Supplementary Fig. (2). Graph showing distribution of the most abundant categories of repeat motifs. The x-axis displays repeat motifs, and y-axis shows relative abundance of the particular motif loci per Mb region of the Cyclotella chloroplast genome.

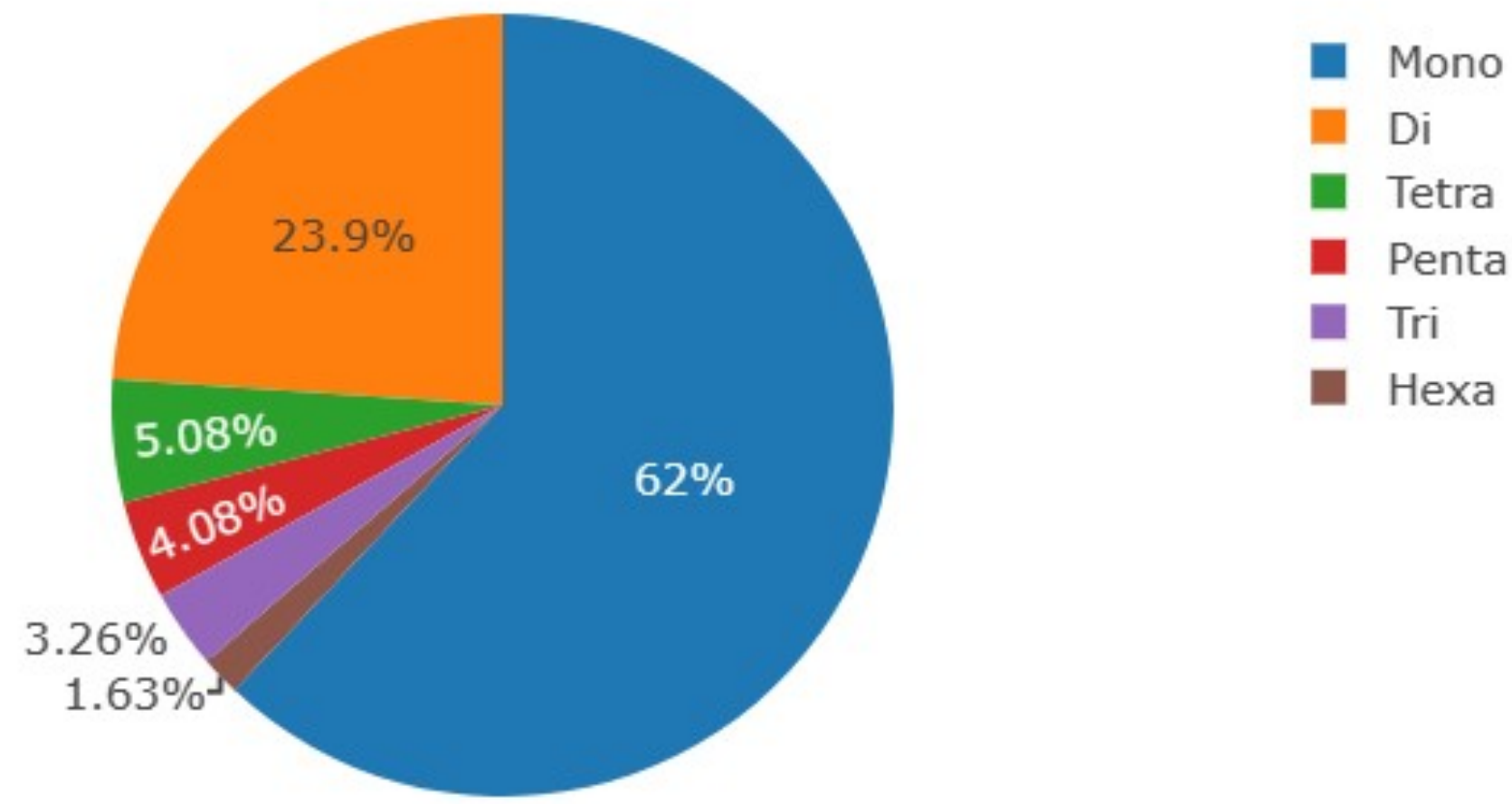

Supplementary Fig. (3). Length-wise representation of SSR identified in the Cyclotella chloroplast genome. Length of each type of SSR represents in terms of percentage of total SSR identified with different colour-coding.
